# The Current Host Range of Hepatitis E Viruses

**DOI:** 10.3390/v11050452

**Published:** 2019-05-17

**Authors:** Scott P Kenney

**Affiliations:** Food Animal Health Research Program, Department of Veterinary Preventive Medicine, The Ohio State University College of Veterinary Medicine, Wooster, OH 44691, USA; kenney.157@osu.edu

**Keywords:** hepatitis E virus, HEV, host range, zoonosis, animals, virus transmission

## Abstract

Hepatitis E virus (HEV) is an emerging zoonotic pathogen transmitting both human to human via the fecal oral route and from animals to humans through feces, direct contact, and consumption of contaminated meat products. Understanding the host range of the virus is critical for determining where potential threats to human health may be emerging from and where potential reservoirs for viral persistence in the environment may be hiding. Initially thought to be a human specific disease endemic to developing countries, the identification of swine as a primary host for genotypes 3 and 4 HEV in industrialized countries has begun a long journey of discovering novel strains of HEV and their animal hosts. As we continue identifying new strains of HEV in disparate animal species, it is becoming abundantly clear that HEV has a broad host range and many of these HEV strains can cross between differing animal species. These cross-species transmitting strains pose many unique challenges to human health as they are often unrecognized as sources of viral transmission.

## 1. Introduction

Hepatitis E virus (HEV) is a single-stranded positive-sense RNA virus thought to be the leading cause of acute viral hepatitis in humans throughout the world [1]. The exact host range of HEV remains muddled, primarily due to the discrete nature of HEV infections. HEV often presents undetectable pathology in infected organisms. Replication levels are typically low and shedding of the virus is sporadic, making the technical and cost to benefit aspects involved in the extensive screening necessary for detecting HEV RNA in all potential host species prohibitive. A single virus serotype among all known strains makes distinguishing infection by differing strains difficult. However, recent data showing newly discovered HEV homologues in fish [2], amphibians [3], moose [4], kestrels [5], and other diverse species suggests the family *Hepeviridae* might possess a host range with similarity to the *Herpesvirales* order which include numerous viruses infecting humans and almost all animal species, including insects, fish, mollusks, reptiles, birds, and mammals [6]. The unique aspects of HEV infection might simply hide its full prevalence. As we continue to evolve more efficient and advanced methods to screen for RNA virus populations and expand our HEV phylogenetic trees, we will inevitably find even more members of the *Hepeviridae* family and their host species, increasing the known host range of the virus.

## 2. Background and Significance

HEV is a substantial human pathogen. In areas where adequate sanitation and clean water are lacking, such as in the developing world or in areas with geopolitical conflicts, there have been numerous instances of large-scale HEV outbreaks involving tens of thousands of individuals [7,8,9]. As our understanding of HEV has expanded, it is now recognized as an emerging zoonotic disease prevalent throughout the world [10,11]. HEV is increasingly being recognized as a threat to immunocompromised populations, including patients receiving organ transplantations [12,13], infected with human immunodeficiency virus [14], battling cancer [15], among others. Understanding the host range of HEV is critical to identifying potential transmission routes to humans, species that serve as reservoirs of viral persistence in the environment, and as potential hosts where HEV can mutate and become even more virulent.

## 3. Hepatitis E Virus Host Range

### 3.1. Factors Determining HEV Host Range

Viral host range is determined by many different factors, both intrinsic and extrinsic. Intrinsic factors to the virus, such as genetic traits encoding advantageous viral proteins, determine its fitness in individual hosts [16]. Intrinsic factors contributing to the host range of HEV include its ability to bind and enter host cells, successful interfacing with the host cell replication machinery, presence of negative regulatory factors, and ability to overcome the host’s innate immune defenses. The ability of HEV to exist as both lipid-associated and naked virus particles likely plays a role in immune system avoidance (enveloped virions) while maintaining stability in the environment (naked virions). The host receptor for HEV remains elusive, possibly complicated by differing entry mechanisms between envelope-associated and naked virions [17]. Without knowing a definitive entry receptor or set of receptors, predicting susceptible hosts through *in silico* modeling is impossible. Host factors necessary for efficient viral replication remain understudied owing to a lack of robust cell culture systems across differing strains [18]. From a mix of in vitro and in vivo data numerous host cellular proteins are known to be altered during HEV infection. These include host proteins involved in metabolism, cholesterol/lipid metabolism, inflammatory/immune responses, and cytoskeleton/trafficking [19]. HEV is reliant upon the host transcription and translational machinery to replicate and efficiently make viral protein [20]. How open reading frame 1(ORF1)—host protein interactions differ between host cells of differing species has not been studied. Unlike known negative host restriction factors in human immunodeficiency virus replication, such as apolipoprotein B mRNA-editing enzyme 3G (APOBEC-3G) or tripartite motif-containing protein 5 (TRIM 5α) and others [21,22,23], similar restriction factors have yet to be assessed for HEV but may contribute to host range. Finally, the host immune response, both innate and adaptive, likely play roles in limiting the host range of HEV. Species capable of mounting functional and lasting immune responses are less likely to serve as reservoir host species, as HEV appears to be particularly susceptible to host-mediated viral clearance with >98% of human cases self-resolving [24]. Extrinsic factors relating to ecology and epidemiology also play significant roles in HEV host range [16]. Examples of extrinsic factors altering HEV host range include the geographic distribution of HEV isolates. Genotype 1 HEV is predominantly spread throughout Africa, Asia, and the Middle East. Genotype 2 is limited to Africa and Mexico. Genotype 3 is the predominant strain circulating in North America, South America, and Europe but also has been found overlapping with genotype 4 in southern and eastern Asia. Genotype 4 HEV is primarily found in Asia with some overlap in Europe. This geographic limitation is also likely a product of immunological cross-protection by predominant circulating strains. For example, if a region has endemic genotype 1 HEV circulating that region is less likely to find genotype 3 or 4 HEV isolates. This phenomenon is likely due to the timing and probability of initial strain exposure coupled with generation of a cross-protective immune response slowing introduction of different strains to that region [25].

### 3.2. Serological Detection

When searching for HEV in new animal species there are two primary methods used for detecting hosts exposed to the virus. Serological testing entails searching for host specific antibodies to the HEV capsid protein utilizing methods such as enzyme linked immunosorbent assays (ELISAs). These assays are thought to work well for broad detection of previous or ongoing HEV infections as there has only been a single serotype attributed to HEV [26,27]. Serological analyses have indicated infections with HEV or HEV-related viruses in a broad range of different animal species. Besides humans, HEV antibodies have been detected in farmed domestic animals, companion animals, laboratory animals, wild animals, and even animals from zoological parks. Serological data does require careful interpretation and should be accompanied by corroborating evidence such as detection of RNA. The presence of only a single serotype of HEV does not allow for designation of the HEV strain to which the animal was exposed without further confirmatory tests. Data presented based solely on seropositivity suggests HEV exposure but is not conclusive. Additionally, HEV ELISA assays have been developed independently in many labs with noted variability between lab groups making data difficult to compare between research groups. Finally, there have been reports of HEV positive ELISA results with the serum containing HEV neutralizing antibodies in the apparent absence of HEV genomes throughout the animal’s life [28]. These results suggest the existence of unknown etiological agents that can generate cross-reacting, HEV-neutralizing antibodies in the absence of HEV infection [28]. Finally, the genetic heterogeneity of HEV does not exclude the possibility that divergent strains may have developed a different serotype that has yet to be detected. 

### 3.3. RNA Detection 

Utilization of reverse transcription polymerase chain reaction (RT-PCR) to directly detect HEV viral genomes is a critical step to diagnosing HEV infections. RT-PCR coupled with DNA sequencing allows the investigator to distinguish between HEV species and strains by genotyping. Simply detecting HEV RNA in fecal or whole organismal samples is also not a definitive indicator of host range or susceptibility. This is evidenced by detection of genotype 3 HEV RNA in mollusks and from vegetables [29,30]. These organisms are not thought to be true hosts for HEV replication but can become contaminated and harbor infectious virus as a potential pass through vector. The ability to detect HEV RNA in host plasma rather than in fecal samples or coupling RNA positive fecal data with HEV antibody seropositivity is a stronger indicator that a host is indeed susceptible to HEV replication. The only three definitive ways to show an organism is truly susceptible to HEV are to look for negative-stranded RNA replication intermediates in infected tissues, to show cell lines derived from the suspect species are capable of replicating HEV, or via experimental infection by showing viremia and virus shedding can persist longer than initial inoculum passing through the host. Many of these methods are very intrusive to the host species rendering them unable to be performed in totality. Hurdles such as necessary tissue samples being difficult to obtain, cell lines unavailable for many animal species, suspect animals themselves are rare and unavailable for experimental infection, and for other reasons. This leads to most of our knowledge relying on the combination of RNA and antibody detection to suggest a species is potentially a host for HEV infection. Frequent and continuous detection of specific HEV types in the same species crossing different geographical areas clearly indicates a true animal reservoir as exemplified by domestic pigs, wild boars, chickens, and rats. In other animal species where HEV is detected sparsely, this suggests spillover infections rather than a true reservoir host. For many animal species, no systematic studies on HEV infections are available, making many of the animals listed throughout Table 1 potential HEV hosts rather than true hosts. A combination of serology, RNA detection, experimental infection, confirmation of viral replication, and multiple detections of HEV in a specified host should be carefully considered before declaring the animal a true host.

### 3.4. Orthohepevirus A

#### 3.4.1. Genotypes 1 and 2 Are Believed to Be Restricted to Higher Primates and Thought to Have Adapted Specifically within Humans

Primate species including rhesus monkeys (*Macaca mulatta*), cynomolgus monkeys (*Macaca cynomolgus*), chimpanzees (*Pan troglodytes*), squirrel monkeys (*Saimiri sciureus*), patas monkeys (*Erythrocebus patas*), Eastern owl monkeys (*Aotus trivergatus*), moustached tamarins (*Saguinus mystax mystax*), and vervet monkeys (*Chlorocebus pygerythrus*) are susceptible to experimental infection with the Sar-55 (genotype 1) and/or Mex-14 (genotype 2) (Table 1). Genotype 1 HEV is seen as the primary circulating HEV strain causing human disease in the developing world with many full-length viral sequences being deposited in Genbank [31]. However, genotype 2 HEV does currently remain in circulation as a threat to human health having been detected in a recent outbreak in Nigeria [32]. How genotypes 1 and 2 HEV remain endemic in the absence of non-primate animal reservoirs remains unknown. To date, only a single report of genotype 1 HEV infecting outside of a primate species exists. Horses (*Equus caballus ferus*) in Egypt had an HEV antibody seroprevalence of 13% and 4% of screened samples were HEV RNA positive. Phylogenetic comparison of a 253-bp gene fragment sequence placed these HEV strains within the genotype 1 lineage [33]. Until more thorough analysis of potential secondary host susceptibility is undertaken, genotypes 1 and 2 HEV appear to be limited to higher primates, except in potentially rare instances where close contact of humans with domesticated animals may lead to some crossover exposure and ability of the virus to pass through hosts, such as horses.

#### 3.4.2. Genotype 3

Genotype 3 is the most well-studied strain of zoonotic HEV. The initial discovery of genotype-3 HEV infecting swine (*Sus scrofa domestica*) within the commercial pork industry completely reshaped the idea of HEV as a solely human pathogen found only infecting humans in underdeveloped countries. Our current understanding of HEV is both as an endemic human pathogen in developing countries and as a zoonotic pathogen spread throughout both the developing and developed world [63,114]. For almost a decade after the discovery of genotype 3 HEV in swine, very few animal hosts were identified. The HEV field focused primarily on primates and porcine hosts as animal reservoirs and as models for studying the disease. Only occasional hints that the HEV host range could potentially span into other species would arise in that decade, such as the serology data for rats [115], dogs (*Canis lupis familiaris*), cattle (*Bos Taurus primigenius*), and rodents [44]. The discovery of an ever-increasing range of potential hosts has begun to emerge as our general understanding of HEV has progressed to its consideration as a serious zoonotic pathogen and the technology to detect HEV infections has grown. Genotype 3 strains cross a significant range of mammalian species from the original identification in domestic pigs (*Sus scrofa domestica*) [63], to a number of species for which humans rely on as agriculturally important species including goats (*Capra hircus aegagrus*) [68,69], sheep (*Ovis aries orientalis*) [41,68], rabbits (*Oryctologus cuniculus*) [66,89,90,91], and horses (*Equus caballus ferus*) [73]. Genotype 3 strains have also been detected in many wild animal game species such as the wild boar (*Sus scrofa*) [102,116], deer, including sika deer (*Cervus nippon nippon)* [102], Yezo deer (*Cervus nippon yesoensis*) [102,113], Roe deer (*Capreolus capreolus*) [97,99], red deer (*Cervus elaphus*) [96,97], and in wild hares such as the European brown hare (*Lepus europaeus*) [66]. Genotype 3 species have even been noted in such exotic species as the Javan mongoose (*Herpestes javanicus*) [80,81,82] and even bottlenose dolphins (*Tursiops truncatus*) [40].

The recent findings that a genotype 3 HEV has been attributed to infection of a bird, the Himalayan griffon (*Gyps himalayensis*), which was coinfected with Aspergillus [72] suggests that given the right host conditions, genotype 3 HEV can become an opportunistic pathogen even infecting across classes such as Mammalia to Aves. Furthermore, in vitro evidence suggests the hypervariable region within the ORF 1 gene contributes to host range optimization [117]. Additionally, naturally occurring host protein insertions enhance HEV replication in cell lines derived from diverse species [118]. These two pieces of evidence suggest that the actual host range of genotype 3 HEV might be very broad if given the opportunity to infect under the right conditions. As our ability and interest to efficiently screen the viromes of differing species increases, so will the known host range for genotype 3 HEV. Scenarios such as the discovery of genotype 3 rabbit HEV in 2009, proceeding from initial discovery, to recognition as a zoonotic pathogen, and finally development into a beneficial animal model in a short amount of time, may become more commonplace [119]. A current listing of animal species shown to be associated with HEV infection are listed throughout Table 1.

#### 3.4.3. Genotype 4 HEV

Genotye 4 is similar to genotype 3 in that it has proven to be a significant zoonotic pathogen.Unlike genotype 3, genotype 4 HEV primarily appears to be contained to Asia and more recently cases have appeared throughout Europe [120]. Like genotype 3 HEV, genotype 4 was initially discovered in domestic pigs and wild boars which serve as a primary reservoir [116]. Recent work out of China suggests cattle, including yellow cattle (*Bos Taurus primigenius*) [45], Holstein Frisian cattle (*Bos Taurus primigenius*) [46], sheep (*Ovis aries orientalis*) [101], and goats (*Capra hircus aegagrus*) [71,121] can be infected by genotype 4 HEV and that infectious virus can potentially be inserted into the human food chain through meat and milk from these animals. In addition to these domestic animals, tufted deer (*Elaphodus cephalophus*) [73], Reeves’ muntjac (*Muntiacus reevesi*), clouded leopard (*Neofilis nebulosa*) [73], and the Asiatic black bear (*Ursus thibetanus*) [36] have tested positive for genotype 4 HEV. In addition to these mammalian species, genotype 4 HEV was detected in two birds in a zoo setting, the crowned crane (*Balearica regulorum*) and silver pheasant (*Lophura* nycthemera) [36]. Like genotype 3 HEV, genotype 4 appears to have a broad host range and may infect many different hosts given appropriate opportunity.

#### 3.4.4. Genotypes 5 and 6 HEV

Genotypes 5 and 6 were isolated from wild boar samples in Japan [108,122]. Recent studies have shown that the virus derived from a genotype 5 infectious clone could cause infections in cynomolgus monkeys that were seronegative for HEV [59]. This research suggests that at least genotype 5 HEV is likely a zoonotic threat to humans and suggests genotype 6 should be evaluated for its ability to infect primates.Human screening is also necessary to confirm their susceptibility. The relatively low circulation of the genotype 5 and 6 virus and geographic seclusion to Japan currently makes transmission to humans a rare occurrence.

#### 3.4.5. Genotypes 7 and 8 HEV

Genotypes 7 and 8 are recently discovered HEV strains whose natural reservoirs are dromedary (*Camelus dromedaries*) and bactrian (*Camelus bactrianus*) camels, respectively [123]. The discovery of genotype 7 HEV chronically infecting a human liver transplant patient demonstrates the ability of this virus to infect humans [77]. Additionally, genotype 8 HEV positive samples were used to infect cynomolgus macaques which were susceptible to both acute and chronic infection [60]. Genotypes 7 and 8 should therefore be considered potential human pathogens with studies screening for genotype 8 in humans still necessary. 

### 3.5. Orthohepevirus B 

*Orthohepevirus B*, or avian HEV, isolates were first identified as causing hepatic splenomegaly syndrome or big liver and spleen disease in chickens [124]. The avian HEV genome shares ~48% identity with mammalian HEVs [124]. At least four different genotypes of avian HEV have been identified from chickens worldwide: genotype 1 from chickens in Australia, genotype 2 from chickens in the USA, genotype 3 from chickens in Europe and China, and genotype 4 from chickens in Hungary and Taiwan [124,125,126,127,128,129]. An infectious clone of genotype 2 avian HEV was created [130] and has served as an important in vivo animal model system for dissecting mechanisms contributing to HEV replication and immunology and to empirically test host susceptibility. Studies with genotype 2 avian HEV showed it could infect turkeys (*Meleagris gallopavo*) but not rhesus monkeys or pigs [131]. A recent publication shows that in addition to turkeys; ducks, geese, and rabbits in mixed housing could be infected by genotype 3 avian HEV, raising concerns that some avian HEV strains could cross into mammalian hosts [132]. Of great concern to the commercial poultry industry is discovery of avian HEV strains that appear to correlate with enhanced pathology in chicks causing hepatic hemorrhage rupture syndrome in China [133]. More avian strains of HEV are continuing to be discovered in expanded bird species. Avian HEV isolates have now been discovered in the little egret (*Egretta garzetta*) with 60–70% identity to genotype 1 avian HEV, the little owl (*Athene noctua*), song thrush (*Turdus philomelos*) [134] and feral pigeon (*Columba livia domestica*) [135]. Even in the United States a sparrow HEV was discovered and showed higher similarity to chicken HEV strains (71–78% and 80% identity) than to little egret HEV (55% and 68% identity) [136]. The host range of *Orthohepevirus B* remains an emerging field. Currently reported susceptible hosts are listed in Table 2. With more thorough testing of differing avian species potentially many more avian HEV strains and hosts will be discovered.

### 3.6. Orthohepevirus C

*Orthohepevirus C* HEV strains were initially detected around the same time as the discovery of swine HEV in 1999 when it was observed that more than 50% of rats within the United States tested positive for anti HEV antibodies [115,138], however, none tested positive for RNA. Lack of HEV RNA was likely due to PCR primer designs based on known *Orthohepevirus A* sequences and due to the sequence divergence of *Orthohepevirus C* from *Orthohepevirus A* strains. More thorough detection methods with broad spectrum PCR primers allowed for detection and eventually sequencing of full-length viral isolates of rat HEV [139,140]. These isolates had approximately 50% or less sequence identity to human and avian HEV strains. Since the discovery of the first sequences of rat HEV, *Orthohepevirus C* strains have been isolated from many different rat species [139,140,141], mice [142,143], greater bandicoot [134], Asian musk shrews [144], ferrets [145], voles [142,146], and mink [67,147] (Table 3). RNA from *Orthohepevirus C*-like strains have also been detected in feces from a red fox (*Vulpes vulpes*) [148], the common kestrel (*Falco tinnunculus*), and red-footed falcon (*Falco vespertinus*) [5]. Whether the fox, kestrel, or falcon-associated *Orthohepevirus C* HEV strains truly infect these host animals or simply pass through from consumed prey, remains debatable. Originally thought as unlikely to pose a threat to infection of higher ordered primates and humans, the recent discovery of a rat HEV strain causing chronic liver disease in a human liver transplant patient [149] greatly increases the urgency to understand this emerging human pathogen. The discovery of zoonotic potential for *Orthohepevirus C*, which had previously not been able to infect rhesus monkeys [150] experimentally, suggests that greater care is necessary to fully vet infectious potential of newly discovered HEV strains and advocates for revisiting potential host ranges of previously tested HEV strains.

### 3.7. Orthohepevirus D

Bat hepatitis E virus shares approximately 57.4–64.2% identity to human HEV genotypes 1–4 [156,157]. Bat HEV has been detected in bats from the *Hipposideridae*, *Phyllostomidae*, and *Vespertilionidae* families [156,157,158] (Table 4). Although one full-length bat HEV sequence exists (JQ001749), no infectious clones currently exist. Bat HEV is not thought to transmit to humans due to its sequence divergence from human infecting strains. In addition, over 93,000 pooled human blood donations screened negative for bat HEV antibodies or RNA [157]. Further research, including an infectious clone coupled with experimental animal infections, is necessary to determine the full host range of bat HEV. 

### 3.8. Piscihepevirus

To date the most divergent strain of HEV, cutthroat trout virus (CTV), shares approximately 40% nucleotide identity with genotype 1 HEV, necessitating phylogenetic classification into its own genus, *Piscihepevirus*, in the *Hepeviridae* family [2]. CTV has been found in Cutthroat trout (*Oncorhynchus clarkii*), rainbow trout (*Oncorhynchus mykiss*), brown trout (*Salmo trutta*), brook trout (*Salvelinus fontinalis*), golden trout (*Oncorhynchus aguabonita*), apache trout (*Oncorhynchus apache*), and gila trout (*Oncorhynchus gilae*) and in a single Atlantic salmon (*Salmo salar*) [160]. Propagation in the CHSE-214 Chinook salmon embryo cell line suggests other salmon and fish species may harbor CTV or related Hepeviruses. The ability of CTV to infect species outside of fish is thought to be unlikely but has not been directly assessed.

## 4. Conclusions

The host range of HEV is broad and continues to expand as technology and funding allows scientists to continually scan new species for this elusive RNA virus. Careful examination of HEV serological data and correlation of results with confirmatory tests such as viremia, presence of negative-strand RNA replication intermediates, or experimental infection studies should all be considered best practice approaches to identifying true hosts for HEV infection rather than suboptimal or pass-through vector hosts.

## Figures and Tables

**Table 1:** Host Range for *Orthohepevirus A*.

Animals Susceptible to Orthohepevirus A Infection					
Animal Species	Scientific Name	Genotype	Serology/Genome Detection^a^	Infection Type	References
	Order				
African green monkey	*Chlorocebus sabaeus*	HEV 1-2	+/ND	Experimental	Doceul et al. [34]
	**Primate**				
American bison	*Bison bison*	?^b^	+/-	Natural	Dong et al. [35]
	**Artiodactyla**				
Asiatic black bear	*Ursus thibetanus*	HEV 4	ND/+	Natural	Zhang et al. [36]
	**Perissodactyla**				
Bactrian camel	*Camelus bactrianus*	HEV 8	+/+	Natural	Woo et al. [37], Rasche et al. [38]
	**Artiodactyla**				
Bonnet macaque	*Macaca radiata*	HEV 1	+/+	Natural	Arankalle et al. [39]
	**Primate**				
Bottlenose dolphin	*Tursiops truncatus*	HEV 3	+/+	Natural	Montalvo Villalba et al. [40]
	**Cetacea**				
Cape buffalo	*Syncerus caffer*	? ^b^	+/ND	Natural	El-Tras et al. [41]
	**Artiodactyla**				
Cat	*Felis catus silvestris*	? ^b^	+/-	Natural	Liang et al. [42], Mochizuki et al. [43]
	**Carnivora**				
Dairy cattle, yellow, Holstein Frisian	*Bos Taurus primigenius*	HEV 4, ? ^b^	+/+	Natural	Arankalle et al. [44], El-Tras et al. [41], Yan et al. [45], Huang et al. [46]
	**Artiodactyla**				
Chimpanzee	*Pan troglodytes*	HEV 1-4	+/+	Experimental Natural	Yu et al. [47], Arankalle et al. [48], Meng et al. [49], Yugo et al. [50], Zhou et al. [51], Spahr et al. [52]
	**Primate**				
Clams (Yamato-shijimi)	*Corbicula japonica*	HEV 3	ND/+	Natural	Li et al. [53]
	**Veneroida**				
Clouded leopard	*Neofelis nebulosa*	HEV 4	ND/+	Natural	Zhang et al. [36]
	**Carnivora**				
Crowned crane	*Balearica regulorum*	HEV 4	ND/+	Natural	Zhang et al. [36]
	**Gruiformes**				
Cynomolgus macaque	*Macaca fascicularis*	HEV 1-5, 8	+/+	Experimental Natural	Balayan et al. [54], Tsarev et al. [55], Bradley et al. [56], Aggarwal et al. [57], de Carvalho et al. [58], Li et al. [59], Wang et al. [60]
	**Primate**				
Dog	*Canis lupis familiaris*	HEV 4? ^b^	+/-	Natural	Liu et al. [61], Liang et al. [42], McElroy et al. [62], Arankalle et al. [44]
	**Carnivora**				
Domestic Pig	*Sus scrofa domestica*	HEV 3, 4	+/+	Experimental Natural	Meng et al. [63],
	**Artiodactyla**				
Donkey	*Equus africanus*	HEV 3	ND/+	Natural	Garcia-Bocanegra et al. [64]
	**Perissodactyla**				
Dromedary camel	*Camelua dromedarius*	HEV 7	+/+	Natural	Woo et al. [37], Rasche et al. [38]
	**Artiodactyla**				
Eastern owl monkey	*Aotus trivirgatus*	HEV 1, 2	+/+	Experimental	Yugo et al. [40], Ticehurst et al. [65]
	**Primate**				
European brown hare	*Lepus europaeus*	HEV 3? ^b^	+/-	Natural	Hammershmidt et al. [66]
	**Lagomorpha**				
Farmed mink	*Neovison vison*	HEV 3	ND/+	Natural	Xie et al. [67]
	**Carnivora**				
Goat	*Capra hircus aegagrus*	HEV 3, 4	+/+	Natural	Peralta et al. [68], El-Tras et al. [41], Sanford et al. [69], Di Martina et al. [70], Li et al. [71], Long et al. [70]
	**Artiodactyla**				
Gray langur	*Semnoppithecus entellus*	HEV 1	+/+	Natural	Arankalle et al. [39]
	**Primate**				
Himalayan griffon	*Gyps himalayensis*	HEV 3	ND/+	Natural	Li et al. [72]
	**Accipitriformes**				
Horse	*Equus caballus ferus*	HEV 1,3	+/+	Natural	Saad et al. [33], Zhang et al. [73]
	**Perissodactyla**				
Human	*Homo sapiens*	HEV 1-4, 7	+/+	Natural Experimental	Balayan et al. [54], Arankalle et al. [74], Huang et al. [75], Meng et al. [63], Hsieh et al. [76], Lee et al. [77]
	**Primate**				
Japanese macaque	*Macaca fuscata*	HEV 3	+/+	Natural	Yamamoto et al. [78]
	**Primate**				
Japanese white rabbit	*Oryctologus cuniculus domesticus*	HEV 3	ND/+	Experimental Natural	Xia et al. [79]
	**Lagomorpha**				
Javan mongoose	*Herpestes javanicus*	HEV 3	+/+	Natural	Li et al. [80], Nakamura et al. [81], Nidaira et al. [82]
	**Carnivora**				
Mongolian gerbil	*Meriones unguiculatus*	HEV 4	+/+	Experimental	Liu et al. [83]
	**Rodentia**				
Moustached tamarin	*Saguinus mystax mystax*	HEV 1,2	+/+	Experimental	Bradley et al. [56]
	**Primate**				
Mussels (Blue mussel, Mediterranean mussel, Pacific mussel),	*Mytilus edulis, Mytilus galloprovincialis, Crassostrea gigas*	HEV 3	ND/+	Natural	O’Hara et al. [84], Krog et al. [85], Diez-Valcarce et al. [86], Crossan et al. [30]
	***Ostreoida***				
Norwegian rat	*Rattus norvegicus*	HEV 3	+/+	Natural	Kanai et al. [87], Lack et al. [88]
	**Rodentia**				
Patas monkey	*Erythrocebus patas*	HEV 1, 2	ND/+	Experimental	Yugo et al. [50]
	**Primate**				
Rabbit New Zealand White, Rex, Japanese White	*Oryctolagus cuniculus* *domesticus*	HEV 3	+/+	Experimental Natural	Cossaboom et al. [89], Izopet et al. [90], Carusoet al. [91], Hammerschmidt et al. [66], Birke et al, [92], Zhao et al. [93], Genget al. [94], Xia et al. [79]
	**Lagomorpha**				
Raccoon	*Procyon lotor*	? ^b^	+/-	Natural	Dähnert et al. [95]
	**Carnivora**				
Raccoon dog	*Nyctereutes procyonoides*	? ^b^	+/-	Natural	Dähnert et al. [95]
	**Carnivora**				
Red deer	*Cervus elaphus*	HEV 3	-/+	Natural	Forgách [96] et al, Anheyer-Behmenburg et al. [97]
	**Artiodactyla**				
Reeves’ muntjac	*Muntiacus reevesi*	HEV 4	ND/+	Natural	Zhang et al. [73]
	**Artiodactyla**				
Rhesus macaque	*Macaca mulatta*	HEV 1-4	+/+	Experimental Natural	Arankalle et al. [39], Yamamoto et al. [78], Meng et al. [49], Huang et al. [98]
	**Primate**				
Roe deer	*Capreolus capreolus*	HEV 3	-/+	Natural	Reuter et al. [99], Forgách et al. [96] 2010, Anheyer-Behmenburg et al. [97]
	**Artiodactyla**				
Sheep	*Ovis aries orientalis*	HEV 3, 4	+/+	Natural	El-Tras et al. [41], Peralta et al. [68], Sarchese et al. [100], Wu et al. [101]
	**Artiodactyla**				
Sika deer	*Cervus nippon nippon*	HEV 3,4	+/+	Natural	Sonoda et al. [102], Zhang et al. [73]
	**Artiodactyla**				
Silver pheasant	*Lophura nycthemera*	HEV 4	ND/+	Natural	Zhang et al. [36]
	**Galliformes**				
Squirrel monkey	*Saimiri sciureus*	HEV 1,2	+/+	Experimental	Tsarev et al. [55]
	**Primate**				
Swedish moose	*Alces alces*	HEV ? ^c^	+/+	Natural	Lin et al. [103], Lin et al. [4]
	**Artiodactyla**				
Tufted deer	*Elaphodus cephalophus*	HEV 4	ND/+	Natural	Zhang et al. [73]
	**Artiodactyla**				
Vervet monkey	*Chlorocebus pygerythrus*	HEV 1,2	ND/+	Experimental	Tsarev et al. [55]
	**Primate**				
Wild boar	*Sus scrofa*	HEV 3,4,5,6	+/+	Natural	Sonoda et al. [102], Martelli et al, De Deus et al. [104], Adlhoch et al. [105], Wiratsudakulet al. [106], Kaci et al. [107], Dong et al. [35], Takahashi et al. [108], Takahashi et al. [109], Larska et al. [110], Carpentier et al. [111], Anheyer-Behmenburg et al. [97]
	**Artiodactyla**				
Yak	*Bos grunniens*	HEV 4	+/+	Natural	Xu et al. [112]
	**Artiodactyla**				
Yezo deer	*Cervus nippon yesoensis*	HEV 3 or 4? ^b^	+/-	Natural	Sonoda et al. [102], Tomiyama et al. [113]
	**Artiodactyla**				

Table 1. Animals known to be associated with *Orthohepevirus A* infection. Text in bold indicates taxonomic order. For serology/genome detection “+” is any reported positive result, “-“ indicates assays were performed and results were negative, “ND indicates test was not performed (Not Done).” ^a^ Serology and genomic RNA detection are a summary of all published data. Individual articles may have only included serology, only genomic RNA detection, or both. ^b^ Data presented as only serological data suggest animals were exposed to HEV but a definitive strain could not be assigned due to the single serotype of all HEV strains. ^c^ Swedish moose HEV strain has not been assigned to a current HEV genotype and appears to cluster between *Orthohepevirus A* and *Orthohepevirus C* strains.

**Table 2:** Host range of *Orthohepevirus B*.

Animals Susceptible to *Orthohepevirus B*					
Animal Species	Scientific Name	Species	Serology/Genome Detection	Infection Type	References
	Order				
Chicken	*Gallus gallus*	Ortho B	+/+	Natural Experimental	Haqshenas et al. [124] Liu et al. [132]
	**Galliformes**				
Common buzzard	*Buteo buteo*	Ortho B	ND/+	Natural	Zhang et al. [135]
	**Accipitriformes**				
Ducks	*Anas platyrhynchos^a^*	Ortho B	+/+	Natural	Liu et al. [132]
	**Anseriformes**				
Feral pigeon	*Columba livia domestica*	Ortho B	ND/+	Natural	Zhang et al. [135]
	**Columbiformes**				
Geese	*Anser anser domesticus^a^*	Ortho B	+/+	Natural	Liu et al. [132]
	**Anseriformes**				
Little egret	*Egretta garzetta*	Ortho B	ND/+	Natural	Reuter et al. [137]
	**Pelicaniformes**				
Little owl	*Athene noctua*	Ortho B	ND/+	Natural	Zhang et al. [135]
	**Strigiformes**				
Rabbit New Zealand White	*Oryctolagus cuniculus*	Ortho B	+/+	Natural Experimental	Liu et al. [132]
	**Lagomorpha**				
Song thrush	*Turdus philomelos*	Ortho B	ND/+	Natural	Zhang et al. [135]
	**Passeriformes**				
Sparrow	*Passer domesticus*	Ortho B	ND/+	Natural	Yang et al. [136]
	**Passeriformes**				
Turkey	*Meleagris gallopavo*	Ortho B	+/+	Experimental	Sun et al. [131]
	**Galliformes**				

Table 2 Animals associated with *Orthohepevirus B* infection. Names in bold represent taxonomic order. Under serology/genome detection “+” indicates any positive report for that assay within the literature, “-“ represents the test being performed with negative results, ND indicates the test was not performed (Not Done). ^a^ Exact species names are uncertain as tested animals were simply identified as duck and goose. E-mails to corresponding authors requesting clarification were not returned.

**Table 3:** Host range of *Orthohepevirus C*.

Animals Susceptible to *Orthohepevirus C*					
Animal Species	Scientific Name	Species	Serology/Genome Detection^a^	Infection Type	References
	Order				
Asian musk shrew	*Suncus murinus*	Ortho C	+/+	Natural	Guan et al. [144]
	**Eulipotyphla**				
Black Rat	*Rattus rattus* *Rattus rattus hainanus*	Ortho C	+/+	Natural	Li et al. [134], Mulyanto et al. [151], Ryll et al. [152]
	**Rodentia**				
Chevrier’s Field Mouse	*Apodemus chevrieri*	Ortho C	ND/+	Natural	Wang et al. [142]
	**Rodentia**				
Common vole	*Microtus arvalis*	Ortho C	ND/+	Natural	Kurucz et al. [146]
	**Rodentia**				
Delicate vesper mouse	*Calomys tener*	Ortho C	ND/+	Natural	de Souza et al. [143]
	**Rodentia**				
European ferret	*Mustela putorius*	Ortho C	+/+	Natural	Raj et al. [145]
	**Carnivora**				
European mink	*Mustelo lutreola*	Ortho C	ND/+	Natural	Krog et al. [147]
	**Carnivora**				
Farmed mink	*Neovison vison*	Ortho C	ND/+	Natural	Xie et al. [67]
	**Carnivora**				
Greater bandicoot rat	*Bandicota indica*	Ortho C	+/+	Natural	Li et al. [134]
	**Rodentia**				
Hairy-tailed bolo mouse	*Necromys lasiurus*	Ortho C	ND/+	Natural	de Souza et al. [143]
	**Rodentia**				
House Shrew	*Suncus murinus*	Ortho C	ND/+	Natural	He et al. [153]
	**Eulipotyphla**				
Human	*Homo sapiens*	Ortho C	+/+	Natural	Sridhar et al. [149]
	**Primate**				
Norway Rat	*Rattus norvegicus*	Ortho C	+/+	Natural Experimental	Kabrane-Lazizi et al. [115], Easterbrook et al. [138], Johne et al. [140], Johne et al. [154], Purcell et al. [150], Widen et al. [155], He et al. [153]
	**Rodentia**				
Peré David’s Vole	*Eothenomys melanogaster*	Ortho C	ND/+		Wang et al. [142]
	**Rodentia**				
Red fox	*Vulpes vulpes*	Ortho C	ND/+	Natural	Bodewes et al. [148]
	**Carnivora**				
Taiwan rat	*Rattus rattoides losea*	Ortho C	+/+	Natural	Li et al. [134], He et al. [153]
	**Rodentia**				
Tanezumi rat (Asian rat)	*Rattus tanezumi*	Ortho C	ND/+	Natural	He et al. [153]
	**Rodentia**				
Yellow-breasted rat	*Rattus flavipectus*	Ortho C	+/+	Natural	Li et al. [134]
	**Rodentia**				

Table 3 Species reported to be infected with *Orthohepevirus C* strains of HEV. Names in bold represent taxonomic order. Under serology/genome detection “+” indicates any positive report for that assay within the literature, “-“ represents the test being performed with negative results, ND indicates the test was not performed (Not Done). ^a^ Serology/Genome Detection summarizes findings from all literature, some article may have only shown serology positive, genome positive, or both.

**Table 4:** Host range of *Orthohepevirus D*.

Animals Susceptible to *Orthohepevirus D*					
Animal Species	Scientific Name	Species	Serology/Genome Detection	Infection Type	References
	Order				
Aba roundleaf bat	*Hipposideros abae*	Ortho D	ND/+	Natural	Drexler et al. [157]
	**Chiroptera**				
Bechstein’s bat	*Myotis bechsteinii*	Ortho D	ND/+	Natural	Drexler et al. [157]
	**Chiroptera**				
Brown long-eared bat	*Plecotus sacrimontis*	Ortho D	ND/+	Natural	Kobayashi et al. [158]
	**Chiroptera**				
Daubenton’s bat	*Myotis daubentonii*	Ortho D	ND/+	Natural	Drexler et al. [157]
	**Chiroptera**				
Great stripe-faced bat	*Vampyrodes caraccioli*	Ortho D	ND/+	Natural	Drexler et al. [157]
	**Chiroptera**				
Japanese short-tailed bat	*Eptesicus japonensis*	Ortho D	ND/+	Natural	Kobayashi et al. [158]
	**Chiroptera**				
Serotine bat	*Eptesicus serotinus*	Ortho D	ND/+	Natural	Drexler et al. [157]
	**Chiroptera**				
Whiskered bat	*Myotis davidii*	Ortho D	ND/+	Natural	Wang et al. [159]
	**Chiroptera**				
					

Table 4 Species susceptible to *Orthohepevirus D* infection. Names in bold represent taxonomic order. Under serology/genome detection “+” indicates any positive report for that assay within the literature and “ND” indicates the test was not performed (Not Done).
